# Delayed cerebral atrophy after cerebellar stroke: topographical relation and clinical impact

**DOI:** 10.1093/braincomms/fcab279

**Published:** 2021-11-24

**Authors:** Christiaan H B van Niftrik, Thomas F Visser, Martina Sebök, Giovanni Muscas, Mohamad El Amki, Carlo Serra, Luca Regli, Susanne Wegener, Jorn Fierstra

**Affiliations:** 1 Department of Neurosurgery, University Hospital Zurich, University of Zurich, 8091 Zurich, Switzerland; 2 Clinical Neuroscience Center, University Hospital Zurich, University of Zurich, 8091 Zurich, Switzerland; 3 Department of Neurology and Neurosurgery, Brain Center Rudolf Magnus, UMC Utrecht, 3584 CX Utrecht, The Netherlands; 4 Department of Neurosurgery, Careggi Hospital and University of Florence, 50134 Florence, Italy; 5 Department of Neurology, University Hospital Zurich, University of Zurich, 8091 Zurich, Switzerland

**Keywords:** cerebellar stroke, cortex, atrophy, diaschisis, clinical outcome

## Abstract

Remote dysconnectivity following cerebellar ischaemic stroke may have a negative impact on supratentorial brain tissue. Since the cerebellum is connected to the individual cerebral lobes via contralateral tracts, cerebellar lesion topography might determine the distribution of contralateral supratentorial brain tissue changes. We investigated (i) the occurrence of delayed cerebral atrophy after cerebellar ischaemic stroke and its relationship to infarct volume; (ii) whether cerebellar stroke topography determines supratentorial atrophy location; and (iii) how cortical atrophy after cerebellar stroke impacts clinical outcome. We performed longitudinal volumetric MRI analysis of patients with isolated cerebellar stroke from the Swiss Stroke Registry database. Stroke location and volume were determined at baseline MRI. Delayed cerebral atrophy was measured as supratentorial cortical volumetric change at follow-up, in contralateral target as compared to ipsilateral reference-areas. In patients with bilateral stroke, both hemispheres were analysed separately. We obtained maps of how cerebellar lesion topography, determines the probability of delayed atrophy per distinct cerebral lobe. Clinical performance was measured with the National Institutes of Health Stroke Scale and modified Rankin Scale. In 29 patients (age 58 ± 18; 9 females; median follow-up: 6.2 months), with 36 datasets (7 patients with bilateral cerebellar stroke), delayed cerebral atrophy occurred in 28 (78%) datasets. A multivariable generalized linear model for a Poisson distribution showed that infarct volume (milliliter) in bilateral stroke patients was positively associated with the number of atrophic target areas (Rate ratio = 1.08; *P* = 0.01). Lobe-specific cerebral atrophy related to distinct topographical cerebellar stroke patterns. By ordinal logistic regression (shift analysis), more atrophic areas predicted higher 3-month mRS scores in patients with low baseline scores (baseline score 3–5: Odds ratio = 1.34; *P* = 0.02; baseline score 0–2: OR = 0.71; *P* = 0.19). Our results indicate that (i) isolated cerebellar ischaemic stroke commonly results in delayed cerebral atrophy and stroke volume determines the severity of cerebral atrophy in patients with bilateral stroke; (ii) cerebellar stroke topography affects the location of delayed cerebral atrophy; and (iii) delayed cerebral atrophy negatively impacts clinical outcome.

## Teaser

Van Niftrik et al. show that cerebellar stroke commonly results in delayed cerebral atrophy and that stroke volume determines the severity of atrophy in patients with bilateral stroke. Furthermore, they observed that cerebellar stroke topography affects the location of cerebral atrophy and that delayed cerebral atrophy negatively impacts clinical outcome.

## Graphical Abstract

**Graphical Abstract:**
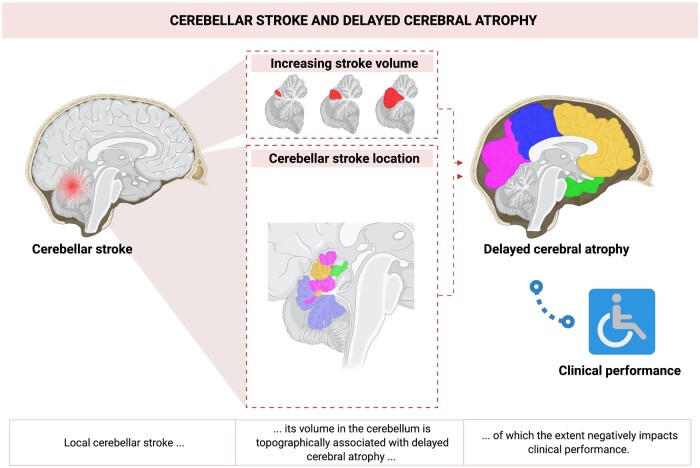

## Introduction

Remote functional dysconnectivity and brain tissue changes following ischaemic stroke remain poorly understood,^1–3^ in particular the remote consequences of isolated cerebellar ischaemic stroke.

**Figure 1:**
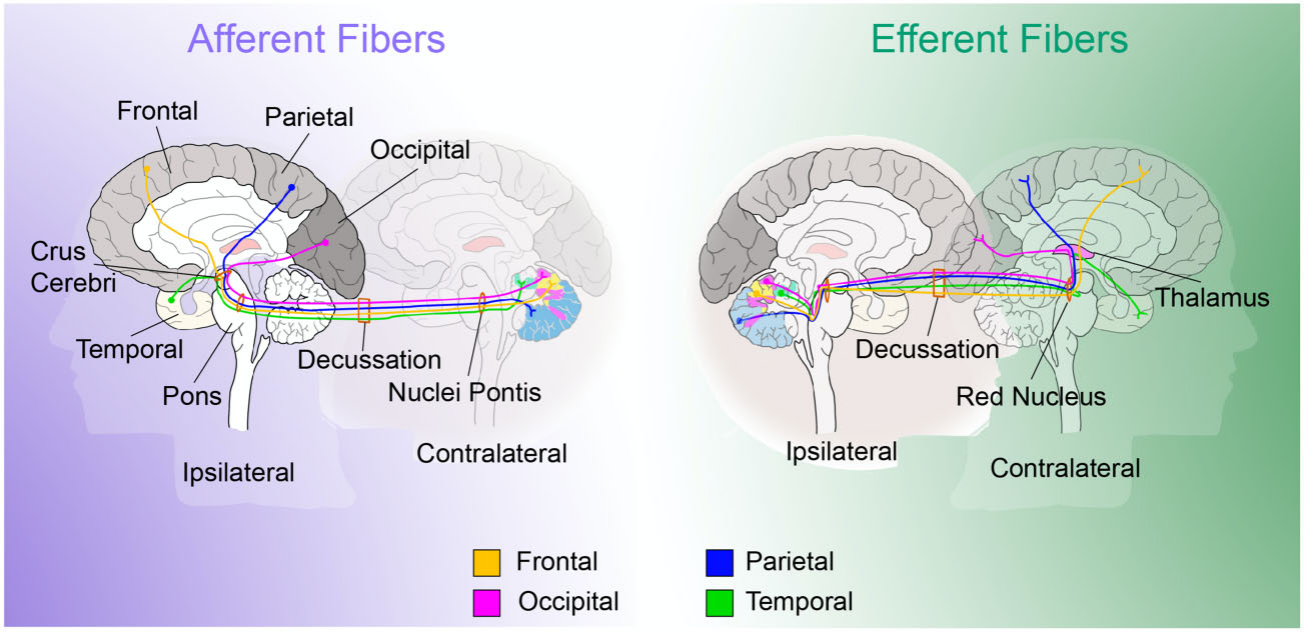
Schematic representation of decussating lobewise cerebrocerebellar tracts. Lobewise afferent cerebro-pontine-cerebellar fibres and efferent cerebello-rubro-thalamic fibres, as connected to their cerebellar representation on the combined probability map. Frontal (orange); Occipital (magenta); Parietal (blue); Temporal (green). From the cerebral cortex, the cerebro-pontine-cerebellar fibres descend thought the ipsilateral crus cerebri to reach the basal pontine nuclei, after which they cross to the contralateral side and enter the cerebellum. The cerebello-rubro-thalamic-cerebral fibres leave the cerebellum, decussate in the caudoventral mesencephalon and are relayed to the cerebral lobes by the nucleus ruber and thalamus.^47^^,^^48^

Previous investigations of crossed cerebellar diaschisis following supratentorial ischaemic stroke have provided valuable insights into the functional properties of the cerebrum–cerebellum interaction.^2^^,^^4^^,^^5^ For instance, crossed cerebellar diaschisis results in hypoperfusion and hypometabolism of the crossed cerebellar hemisphere,^1^^,^^2^^,^^4^ whereas the recruitment of blood flow, i.e. the blood flow reserve capacity, of the affected cerebellar hemisphere remains intact.^4^ These remote cerebellar neurophysiological effects indicate a neuronal deactivation of the cerebro-pontine-cerebellar or cerebello-rubro-thalamic-cerebral tracts, through which the cerebrum is connected to the cerebellum via different lobe-wise sub-circuits (Fig. 1).^6–10^ This results in a rather ‘hibernating’ cerebellar hemisphere without apparent acute tissue injury or clinical signs of cerebellar dysfunction, although these patients appear to develop cerebellar atrophy and have a worse clinical outcome.^1^^,^^11^^,^^12^

Potentially, in a reversed manner, cerebellar disruption of these tracts may as well cause supratentorial changes, indicating cerebello-cerebral diaschisis as an inverse equivalent of crossed cerebellar diaschisis.^13^ In line with this hypothesis, recent investigations have reported attenuated metabolism and blood flow of the contralateral supratentorial cortex after isolated cerebellar ischaemic stroke as possible signs of a cerebello-cerebral diaschisis.^14–17^ Whether such a disruption impacts supratentorial structural integrity i.e. a cerebellar stroke leading to delayed supratentorial brain atrophy, remains unknown. Since the cerebellum is connected to the individual cerebral lobes via contralateral tracts, cerebellar lesion topography might determine the distribution of contralateral supratentorial brain tissue changes.

The aim of this study was to assess whether delayed cortical atrophy can be found in patients with isolated cerebellar ischaemic stroke. As the aforementioned lobe-wise sub-circuits may determine specific locations of supratentorial brain areas amendable to atrophy, we aim to locate specific cerebellar regions corresponding to lobe-wise supratentorial volume loss. Last, we will investigate whether the extent of supratentorial cortical atrophy impacts clinical outcome.

## Methods

### Patient selection

Patients were selected from the ongoing prospective Swiss Stroke Registry database according to procedures approved by the Zurich cantonal ethics committee and in accordance with the Declaration of Helsinki (KEK: ZH2012-0427). The Swiss Stroke Registry database contains information about all patients with suspected acute cerebral ischaemia or haemorrhage admitted to the Clinical Neuroscience Center of the University Hospital Zurich as of January 2013. At the time of patient screening for this study, i.e. October 2018, the database contained 3443 ischaemic stroke patients.

Patients were retrospectively included based on the following criteria: An isolated unilateral or bilateral cerebellar ischaemic stroke lesion visible on diffusion-weighted imaging (DWI), and a baseline cerebral MRI scan as well as at least one follow-up MRI scan. A baseline MRI was defined as an MRI scan within 1 week after onset of clinical symptoms. The follow-up MRI scan needed to be done at least 3 weeks after the initial imaging, since this represents the minimal time known to develop remote structural changes within the central nervous system.^18^ MRI datasets of patients without a DWI scan at baseline and without an anatomical high-resolution T_1_-weighted scan at baseline or follow-up were excluded. Patients with cerebellar haemorrhagic stroke, cerebellar lesions from another entity such as a neoplastic or vascular type, a history of other neurological diseases and patients with new lesions detected on follow-up MRI were excluded as well. On admission, none of the included study patients exhibited signs of supratentorial hypoperfusion on either a Computer Tomography perfusion imaging or Magnetic Resonance perfusion imaging.

### Segmentation of cerebellar ischaemic stroke lesion

The location of the acute cerebellar infarctions was derived from the baseline DWI scan of each patient and manually outlined using MRIcron software (NeuroImaging Tools & Resources Collaboratory, University of South Carolina, 2008, https://www.nitrc.org/projects/mricron). The resulting stroke mask was then registered to the anatomical T_1_-weighted imaging volume and normalized in Montreal Neurological Institute (MNI) space of the MNI/ICBM AVG 152 template using a 12-parameter affine linear transformation and warping of the images using a nonlinear transformation using custom algorithms of SPM 12 (Statistical Parameter Mapping software, Welcome Trust Centre for Neuroimaging, Institute of Neurology, University College London; http://www.fil.ion.ucl.ac.uk/spm).

### Topographical cortical surface segmentation to determine supratentorial cortical volume

Volumetric cortical segmentation was performed using Freesurfer 5.3.0 (http://surfer.nmr.mgh.harvard.edu/fswiki/FreeSurferWiki).^19^^,^^20^ In both hemispheres, 74 cortical areas where distinguished using the Destrieux atlas.^21^ Based on this atlas, only areas located in the frontal (*n* = 22), temporal (*n* = 12), occipital (*n* = 14) and parietal (*n* = 12) lobes were taken into analysis. The resulting areas were then classified as either *target* or *reference* area. Cortical areas contralateral to the cerebellar pathology were classified as target areas, whereas cortical areas ipsilateral to the cerebellar pathology were classified as reference areas. Reference areas were obtained only in patients with unilateral cerebellar ischaemic stroke. Target areas in bilaterally affected patients were analysed individually.

### Determining supratentorial cortical atrophy

All computations were done using MATLAB2020a based (The MathWorks, Inc., Natick, United States; www.matworks.com) in-house written scripts.

Data of each reference and target area at baseline and follow-up were converted to *Z*-scores, calculated using their mean and standard deviation at baseline. In this way, a standardized measure of change relative to baseline was obtained per area.

Data of each reference area were then combined across patients to linearly fit the change in the number of standard deviations relative to baseline over time. To account for different numbers of follow-up acquisitions per patient, a mixed effects model was used with subject identity as a random effect. Change in reference areas relative to baseline was fitted without an intercept, as change at baseline is zero by definition.

Next, a Bonferroni corrected 95% prediction interval was computed. In calculating the prediction interval of unilaterally affected patients, conditional model estimations were used, considering fixed as well as random effects. As intra-subject random effects were not estimated for bilaterally affected patients, this prediction interval was based on fixed effects only, using marginal model predictions.

Because remote effects of cerebro-cerebellar disruption are commonly observed in only a subset of patients,^4^ and as we suspected stroke location to cause considerable variation among patients, we avoided group-level statistics of target areas. Therefore, the cortical volumetric change at follow-up in each individual target area was compared to the estimated change in the corresponding reference area. A change beneath the lower prediction interval-limit was considered significant. Target areas showing significant cortical volume loss were considered atrophic target areas. The analysis was repeated with prediction intervals of 90%, 80%, 70%, 60% and 50% to validate our method by assessing the consistency of the main outcomes.

### Cerebellar probability maps of supratentorial lobe-wise atrophy

To obtain cerebellar probability maps of supratentorial lobe-wise cortical volume loss, patients were categorized as having significant frontal, temporal, occipital or parietal cortical volume loss. After categorization, the binarized cerebellar stroke lesion masks of patients in each category were compiled to obtain lobe-wise probability maps. All maps were normalized based on the number of patients in the respective group to obtain percentile probability values, expressing the chance of a cerebellar voxel being affected per lobe-wise group. Following the same procedure, a probability map of general cerebellar stroke location was obtained. Using the cerebellar stroke maps of all cases, we made a map depicting the chance of a voxel being affected in our study population. Next, lobe-wise ratio maps were created by dividing all lobe-wise percentile probability maps by the percentile probability map of stroke location. In this way, the lobe-specific maps were corrected for the variability in stroke location: depicting the relative risk of a cerebellar voxel being affected in lobe-wise groups as compared to the study population. Lobe-wise maps were spatially smoothed using a Gaussian filter [full-width at half-maximum (FWHM) 5 mm].

Finally, a single combined map was obtained by comparing lobe-wise ratio maps. Each cerebellar voxel was assigned to the lobe-wise map showing the highest value. The combined map therefore depicts the supratentorial lobe most likely to show delayed cortical atrophy per affected cerebellar voxel.

### Scores to quantify clinical neurological performance

To assess neurologic state at the time of cerebellar stroke and at 3-months follow-up, the National Institutes of Health Stroke Scale (NIHSS) and modified Rankin Scale (mRS) were used. NIHSS and mRS scores are scores normally used to evaluate the clinical status of patients with anterior circulation strokes and are therefore a suitable surrogate measurement of clinical implications of supratentorial structural changes.^22–24^

### Statistical analysis

The volumetric change in reference areas was determined with the aforementioned mixed model analysis. Furthermore, the effect of baseline cortical volume per reference area on volumetric change over time was assessed with a mixed effects model. In both analyses, a Bonferroni corrected *P*-value of <0.05 was considered significant.

Next, the relationship between volume and the presence of bilateral ischaemic stroke with the number of atrophic target areas was assessed with generalized linear models for a Poisson distribution. This model was chosen as the number of atrophic target areas is a count variable, which commonly follows a Poisson distribution. All models were corrected for potential overdispersion. First, univariable analysis of the association of atrophic target areas with stroke volume, the presence of bilateral stroke, the baseline cortical volume of the target hemisphere, the side of the affected cerebellar hemisphere, follow-up duration, gender and age was performed. Next, multivariable analysis was used to assess the effect of stroke volume, the presence of bilateral stroke and the interaction between both variables on the number of atrophic target areas. These effects were corrected for baseline cortical volume of the target hemisphere, the side of the affected cerebellar hemisphere, follow-up duration, gender and age. Results are reported as rate ratios (RRs).

Next, the association between the number of atrophic target areas and 3-month mRS and NIHSS scores was assessed, in patients with both baseline and follow-up clinical scores available. The relationship between the covariates and 3-month performance was explored using uni- and multivariable ordinal logistic regression analysis, i.e. shift analysis.^25^ As the interaction between atrophic target areas and long-term performance score is most relevant in patients with unfavourable baseline scores, we aimed to assess both categories separately. Therefore, baseline performance scores were dichotomized: Baseline mRS score into zero to two points (functionally independent) and three to five points (impaired self-support). NIHSS scores into below six (none or mild impairment) and six or more points (moderate–severe impairment).^26^ Additional covariates included age, gender and the presence of bilateral stroke. Furthermore, an interaction term between dichotomized baseline score and the number of atrophic target areas was incorporated in the multivariable model. Side of stroke (left or right), baseline cortical volume and stroke volume were not included to prevent potential multi-collinearity. Results were reported as odds ratios (ORs). To test the proportionality of odds assumption, uni- and multivariable binary logistic regression was used to obtain an OR per predictor per outcome level. Concerning the regression analysis, a *P*-value below 0.05 was considered statistically significant.

### Data availability

The data that support the findings of this study are available upon reasonable request from the corresponding author.

## Results

### Patient characteristics

**Figure 2:**
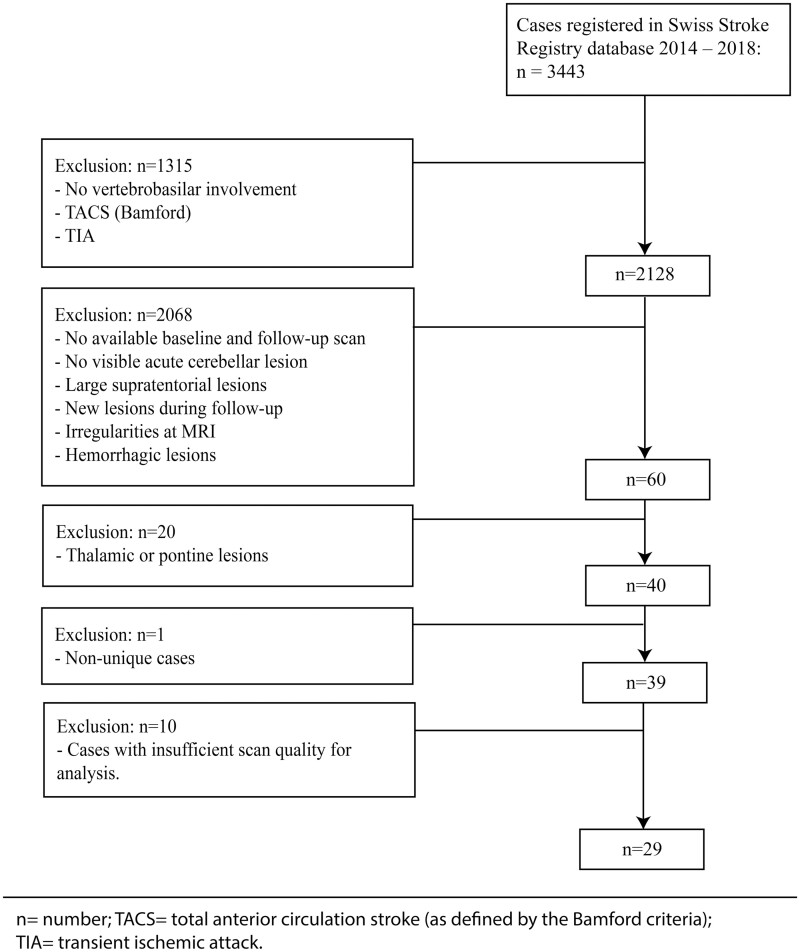
Flow chart depicting patient inclusion. For the final analysis, we included 29 patients providing 36 datasets (seven patients had bilateral cerebellar stroke). *n*, number; TACS, total anterior circulation stroke; TIA, transient ischaemic attack.

A flow-chart depicting the inclusion and exclusion algorithm can be found in Fig. 2. A total number of 29 patients of which seven patients had bilateral cerebellar ischaemic stroke, were included (total number of analysed datasets: *n* = 36). An overview of baseline patient characteristics is given in Table 1.

**Table 1:** Patient characteristics

Patient characteristics	Sample size
	*n* = 29
Age at baseline	
Mean (SD)	58 (18)
Gender	
Female (%)	9 (31%)
Lesion volume (ml)	
Mean (SD)	5.4 (8.7)
Cerebellar lesion lateralization	
Left	11 (38%)
Right	11 (38%)
Bilateral	7 (24%)
Mean follow up time (months)	
Median (IQR)	6.2 (8.4)
Number of follow-up scans (n)	
1	17
2	11
3	1
NIHSS ad admission	
Median (IQR)	2 (3)
NIHSS after 3 months^a^	
Median (IQR)	0 (1)
mRS ad admission	
Median (IQR)	2 (2)
mRS after 3 months	
Median (IQR)	1 (1)

IQR, interquartile range; *n*, number; SD, standard deviation.

a *n* = 27 due to missing data.

### Delayed cerebral atrophy following isolated cerebellar ischaemic stroke

As corrected for the number of 36 datasets, a change below the 99.86% PI of the fitted reference data was considered significant. Atrophic target areas were seen in 28 (78%) datasets. The median number of atrophic target areas per dataset was 1.5 out of 60 (IQR, 2).

Considering the number of 20 reference cases, a *P*-value of 0.0025 was considered significant in assessing the change in each reference area and its relationship to baseline cortical volume. None of the 60 reference areas showed a significant cortical volumetric change between baseline and last follow-up. When including baseline cortical volume as a predictor of change over time, no significant interactions were found. The total number of patients showing cortical atrophy per target area and the baseline cortical volume of all target areas are depicted in Supplementary Fig. 1.

Univariable Poisson regression identified no significant predictors of the number of atrophic target areas (Supplementary Table 1). Multivariable Poisson regression revealed a significant interaction between cerebellar stroke location (bilateral versus unilateral) and stroke volume (RR = 1.14; *P* = 0.02—Table 2). We therefore stratified patients by stroke location, into groups with unilateral and bilateral cerebellar stroke. A significant association between stroke volume and the number of atrophic target areas was observed in patients with bilateral stroke (RR = 1.08, *P* = 0.01).

**Table 2:** Multivariable analysis of the number of atrophic target areas at last follow-up

		RR	95% CI	*P*-value
Stroke location (bilateral)		0.51	0.20–1.30	0.15
Side of stroke (left)		0.91	0.48–1.70	0.75
Baseline cortical volume		1.16	0.98–1.38	0.08
Age		1.02	0.99–1.04	0.23
Follow-up time		0.93	0.87–1.00	0.049*
Volume: stroke location (bilateral)		1.14	1.03–1.26	0.02*
Volume:	unilateral stroke^a^	0.95	0.87–1.03	0.23
	bilateral stroke^a^	1.08	1.02–1.14	0.01*

CI = confidence interval; RR = rate ratio.

*p-values < 0.05.

Units: Volume, ml.; Baseline cortical volume, 10 ml.; Age, years; follow-up time, months.

Predictors of the number of atrophic target areas: The follow-up time, baseline cortical volume and age are normalized to their mean, the corrected effects must therefore be interpreted at their mean respective values.

a Effect of volume on the number of atrophic target areas, in groups stratified by stroke location (bilateral versus unilateral).

This implies that in these patients, a 1 ml higher stroke volume predicts an 8% increase in the number of atrophic target areas, i.e. more cortical atrophy over time. Furthermore, a longer duration between baseline and follow-up scans, showed a significant negative association with the number of atrophic target areas. Lastly, the observed association between stroke volume and the number of atrophic target areas, was consistent across different prediction interval cut-offs for the initial determination of atrophy (Supplementary Table 2).

### Probability maps of supratentorial cortical atrophy

**Figure 3:**
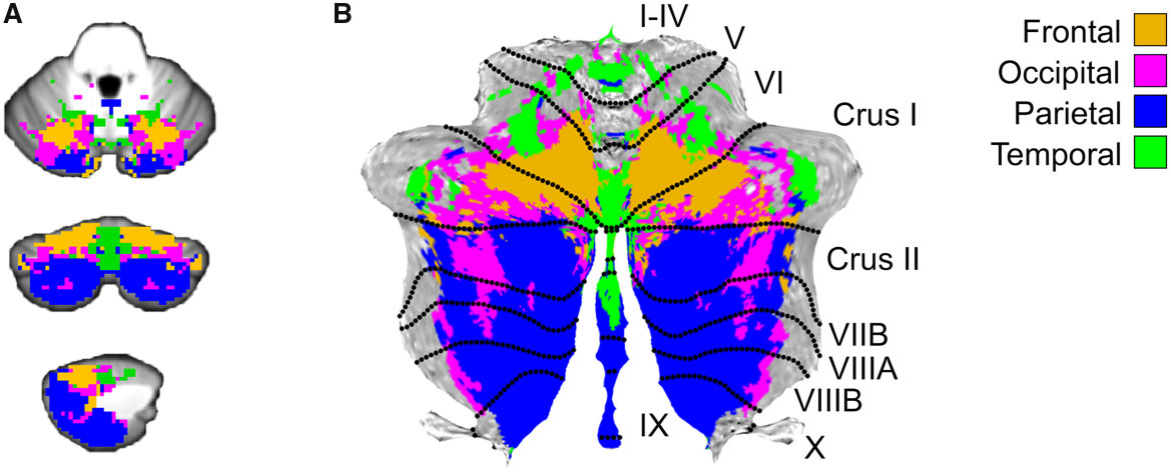
**Combined lobe-wise probability map. Combined lobe-wise probability map. Depicting the supratentorial lobe most likely to show delayed cortical atrophy per affected cerebellar voxel. Frontal (orange); Occipital (magenta); Parietal (blue); Temporal (green).** (**A**) Projected on anatomical cerebellum-only atlas template.^49^ Respectively axial, coronal and sagittal planes. (**B**) Projected on cerebellar grey matter flatmap.^50^

Of 36 datasets, 15 datasets were classified as having frontal, 11 as having occipital, 18 as having parietal and 9 as having temporal tissue volume loss. Lobe-wise probability maps show how cerebellar lesion topography determines the probability of delayed atrophy per distinct cerebral lobe (Supplementary Fig. 2). The combined probability map shows how cerebellar lesion topography determines which cerebral lobe will most likely show delayed atrophy after cerebellar ischaemic stroke (Fig. 3).

### Clinical outcome

Median mRS at admission and after 3 months FU are depicted in Table 1. Between patients with unilateral and bilateral cerebellar ischaemia, no significant differences were seen for both mRS and NIHSS at admission [unilateral versus bilateral: mRS: 2(2) versus 3(1), *P* = 0.08; NIHSS: 2(1) versus 1(2), *P* = 0.89] and after 3 months [mRS: 1(1) versus 1(1), *P* = 0.54; NIHSS: 0(1) versus 0(1), *P* = 0.51].

Univariable analysis revealed baseline mRS as the only significant predictor of 3-month mRS (Supplementary Table 3). The multivariable analysis showed a significant interaction between baseline mRS score (mRS = 0–2 versus mRS ≥3) and the number of atrophic target areas (OR = 1.89; *P* = 0.04—Table 3). We therefore stratified patients by baseline score, into groups with low (mRS = 0–2) and high (mRS ≥3) baseline scores. A significant independent effect of the number of atrophic target areas on 3-month mRS score was found in patients with high baseline mRS values (OR = 1.34; *P* = 0.02). This implicates that in patients with high baseline mRS values, one additional atrophic target area increases the likelihood of having a 1-point higher 3-month score with 34%. Considering the NIHSS score, multivariable analysis revealed that only higher age had a significant positive effect on 3-month NIHSS (OR= 1.14; *P* = 0.01—Supplementary Tables 4 and 5). Some violations of the proportionality of effects assumption occurred, which are described in the supplementary materials (Supplementary text 1). Lastly, the observed association between number of atrophic target areas and mRS score, was consistent across different prediction interval cut-offs for the initial determination of atrophy (Supplementary Table 6).

**Table 3:** Multivariable analysis of 3-month mRS score

		OR	95% CI	*P*-value
Baseline mRS (≥3)		1.63	0.13–23.09	0.71
Stroke location (bilateral)		0.44	0.04–4.55	0.50
Age		1.05	1.00–1.10	0.07
Gender (male)		0.39	0.06–2.31	0.30
Number of atrophic target areas: baseline mRS (≥3)		1.89	1.12–3.84	**0.04**
Number of atrophic target areas	baseline mRS = 0–2^a^	0.71	0.38–1.11	0.19
	baseline mRS ≥ 3^a^	1.34	1.05–1.76	**0.02**

CI = confidence interval; mRS = modified Rankin Scale; OR = odds ratio.

Units: Age, years.

Adjusted odds ratios from multivariable ordinal regression analysis of 3-month mRS score. The patient age is normalized to its mean, corrected effects of stroke location must therefore be interpreted at mean age. All patients had available baseline and follow-up mRS scores.

a Effect the number of atrophic target areas on 3-month mRS score, in groups stratified by baseline mRS (baseline mRS = 0–2 versus baseline mRS ≥3).

As stroke volume had a strong association with the atrophic target areas, it was deemed a confounder and excluded from this analysis.

## Discussion

Our data demonstrate that isolated cerebellar ischaemic stroke commonly results in delayed contralateral supratentorial cortical atrophy. We observed that in case of bilateral cerebellar stroke, the severity of atrophy is determined by cerebellar ischaemic volume. These supratentorial effects of cerebellar stroke may be attributed to disruption of the tracts that contralaterally connect the cerebellum to individual cerebral lobes. By applying a lobe-wise analysis, we found cortical atrophy per cerebral lobe to relate to specific patterns of cerebellar lesion topography. Furthermore, an increased number of atrophic supratentorial cortical regions resulted in poorer clinical outcome.

### Occurrence of delayed supratentorial atrophy after cerebellar stroke

Delayed cerebral brain tissue atrophy was measured as volume loss over time per pre-specified anatomical region (target area) as compared to an intra-subject reference. The common occurrence of supratentorial brain atrophy is line with our hypothesis. However, the suspected influence of infarct volume on the amount of atrophy was observed only in bilaterally affected patients, where target areas are analysed in both cerebral hemispheres separately. The presence of bilateral stroke alone was no significant predictor of the number of atrophic target areas. This confirms that volume is the major determinant of supratentorial atrophy. The bilateral-only effect of volume may be explained by the neural organization of post-stroke brain recovery. It is known that interhemispheric connections have a role in structural recovery after supratentorial stroke.^27^ Furthermore, recovery of functional cerebrocerebellar connectivity after infratentorial stroke is associated with normalization of cerebral interhemispheric connectivity.^28^ Bilateral stroke might prevent contralesional compensation, potentially accentuating the effect of lesion volume on supratentorial atrophy.

Together, these results support the theory that cerebral regions are unilaterally represented in (partially) distinct, unilateral cerebellar regions. Inside these cerebellar regions, infarct lesions have a role in reducing the supratentorial tissue volume of specific supratentorial regions.

### Cerebellar lesion topography and delayed supratentorial atrophy

Based on previous research, we hypothesized that regional probability of supratentorial brain atrophy depends on the distribution of cerebro-cerebellar white matter tracts within the cerebellum. Several studies report a lobe-wise organization of the afferent cerebro-pontine-cerebellar as well as the efferent cerebello-rubro-thalamic-cerebral tracts.^6–9^ Therefore, we created a probability map consisting of lobe-wise pairings: depicting the influence of cerebellar stroke location on atrophy in separate cerebral lobes, by assessing the highest probability of stroke location in patients showing atrophy per cerebral lobe.

The combined map shows the relative dominance of the frontal probability hotspot in the medial portions of lobules VI to crus I and of the parietal hotspot in crus II to lobe IX. The temporal hotspot is mainly bound to the vermis and the medial small regions in lobes I–V. The occipital lobe occupies mostly the frontal-parietal border zones. As change was most often seen in frontal and parietal lobes, this may have influenced the reference map. The occipital borderzone-hotspots might therefore reflect differences to frontal and parietal maps, instead of to the reference map only. It is evident that the hotspots of lobe-wise maps show overlap, possibly explaining inconsistencies between previous tractography studies in characterizing lobar tracts.^7^^,^^17^

Our findings are in line with earlier studies that assessed the topographical representation of structural connectivity. In accordance with the observed hotspots, DTI-tractography has shown that the cerebro-pontine-cerebellar and cerebello-rubro-thalamic-cerebral tracts are primarily derived from lobules VI, VII (CrusI-VIIb) and VIII.^7^^,^^8^ Furthermore, in line with the observed large frontal and parietal hotspots, significant supratentorial metabolic and hemodynamic effects of diaschisis after cerebellar stroke have been observed mainly in the contralateral frontal and parietal lobes.^15^^,^^17^^,^^29^ Furthermore, tractography studies report that the cerebro-cerebellar tracts are restricted mainly to the frontal^7^^,^^8^^,^^30^ and parietal lobes.^30^

In contrast to our results, however, relatively high temporal and lower parietal cerebrocerebellar involvement in the cerebrocerebellar tracts has been reported as well.^7^^,^^8^ It must, however, be noted that reconstruction of temporal cerebrocerebellar tracts remains highly inconsistent among studies,^6^^,^^9^^,^^30^^,^^31^ emphasizing the added value of our unique analysis approach.

### Clinical impact of supratentorial atrophy after cerebellar stroke

We investigated the relationship between supratentorial brain atrophy and neurological function, by assessing the clinical performance reflected by NIHSS and mRS score. Our main finding in respect of clinical outcome is that the number of atrophic target areas predicts a worse mRS score at 3 months. Likewise, the predictive effect of baseline mRS score alone is highly dependent on its modulation by the number of atrophic target areas, as it is only significant without correction for the interaction term. This might indicate indirect supratentorial atrophy as a pathophysiological determinant of clinical outcome.

Insightful to the origin of atrophic target area dependent clinical impairment, is the cerebellar distribution of functions related to the cerebrum specifically. In this regard, fMRI, lesion-symptom mapping and neurodegeneration studies distinguish a sensorimotor versus a cognitive cerebellum. The sensorimotor cerebellum (lobes I–VI, VIIb and VIII) is functionally connected to frontoparietal somatosensory areas.^32–39^ The cognitive cerebellum [crus I and II (lobe VIIa), VIIb and IX] is functionally connected to frontoparietal association areas.^32–35^^,^^37^ The prominent involvement of frontoparietal areas in cerebrocerebellar functions is in line with the large frontal and parietal hotspots we observed. Of further relevance to our study, is the distribution of frontal versus parietal functions specifically. However, making a topographical distinction harder, both frontal and parietal cerebrocerebellar circuits partake in motor as well as cognitive functioning. Nevertheless, relatively anterior cerebellar activation is seen when performing typical frontal functions like repetitive movement and motor planning, while more posterior activation is seen when performing parietal functions like visuomotor coordination and visuospatial cognitive tasks.^35^^,^^37^ This is in accordance with the relative anterior–posterior localization of the respective frontal and parietal hotspots we found. The absence of a hotspot in lobes I–V, might be discrepant from the presumed anterior localization of frontal functions. However, relatively few patients had a lesion in lobes I–V, similar to earlier studies of cerebellar stroke.^40^ This might also explain why the most frequently observed atrophic target areas in our study, like the fronto-marginal volume and parietal angular gyrus, are linked to higher cognitive—instead of primary somatosensory functioning.^41^^,^^42^

We identified temporal and occipital hotspots in the vermal part of lobe VI (Fig. 3 and supplementary Fig. 2). This region contains the cerebellar fastigial nuclei and has been linked to emotion processing, saccadic eye movement and visual processing.^34^^,^^35^^,^^39^^,^^43^ This is in line with temporal lobe functions in emotional processing as well as the occipital and temporal roles in visual processing.^44–46^

To conclude, atrophic-target-area-dependent clinical impairment might originate from the characteristic representation of cognitive and somatosensory functions in distinct frontal, parietal and temporal cerebrocerebellar circuits. Considering the proportion of 78% of datasets with significant supratentorial atrophy, our findings emphasize the clinical relevance of these remote effects in patients with cerebellar ischaemic stroke.

### Limitations

In interpreting our study, it is important to acknowledge some limitations. Our sample was selected in a single tertiary stroke centre, which may limit the generalizability of our results to patients treated in other institutions. Furthermore, due to the retrospective nature of the study, our follow-ups were done at different times and done with scans of different sequences and variable scan quality. We aimed to adjust for this limitation by using the subjects own ipsilateral hemisphere as a reference. Furthermore, we assumed the cerebro-cerebellar connectivity to be contralaterally arranged. Crossed cerebellar diaschisis emphasizes the major relevance of the contralateral cerebro-pontine-cerebellar and cerebello-rubro-thalamic-cerebral tracts in respect of remote structural effects. Ipsilateral tracts however, have also been reported.^31^ Also, we assumed strictly reciprocal cerebro-cerebellar sub-circuits. This premise might be nuanced by considering the integration of information of other cerebellar afferent pathways by, as well as the translation of information between sub-circuits. Moreover, the sensitivity of our method to determine atrophic target areas at long follow-up was relatively low, as few patients in this time range were included. The negative correlation between time until last follow-up and the number of atrophic target areas illustrates this reduced sensitivity. Follow-up duration might therefore have influenced the association between the number of atrophic target areas and 3-month performance score, for which we did not correct to avoid the risk of overfitting the model. consequences of cerebellar stroke. In assessing predictors of clinical performance, our analysis violated the proportionality of odds assumption. Common statistical methods to test this assumption are known to provide unreliable *P*-values. Therefore, we chose a method providing additional information about the extent of violation per predictor per level. However, no unequivocally right criterion to determine violation of the proportionality of odds exists. We therefore accepted the suspected minor violations out of pragmatic consideration, as the alternative, partial ordinal logistic regression, suffers from very limited clinical interpretability.

Last, due to the retrospective nature of this study, the association between supratentorial atrophy and clinical outcomes is of interest, however, remains speculative. Specific factors, may influence this relationship, cannot be reliably determined retrospectively and should therefore be a focus for future studies. Similarly, mRS and NIHSS scores are only general neurological scores of supratentorial neurological functioning and more specific functional or cognitive testing could result in more individual and unambiguous associations, especially in relationship to the brain region affected by delayed atrophy.

However, while acknowledging these limitations, our method is unique in assessing remote volumetric change after cerebellar stroke in the humans, as it surpasses caveats of animal studies as well as MRI tractography. Our method therefore provides new insights in structural cerebral-cerebellar connectivity, which could form the basis of more detailed investigation of the remote effects of cerebellar stroke.

## Conclusion

We demonstrate that isolated cerebellar ischaemic stroke commonly results in delayed cerebral atrophy. In patients with bilateral cerebellar stroke, the severity of atrophy is determined by cerebellar ischaemic volume. Furthermore, we were able to identify specific cerebellar regions that are topographically associated with supratentorial cortical atrophy in distinct cerebral lobes. Finally, an increased number of atrophic cortical regions is associated with poorer clinical outcome.

## Supplementary material

Supplementary material is available at *Brain Communications* online.

## Funding

This project was funded by the Clinical Research Priority Program of the University of Zurich (Clinical Research Priority Program Stroke). Dr Jorn Fierstra was also supported by the Swiss Cancer League (KFS-3975-082016-R). Professor Susanne Wegener was supported by the Swiss National Science Foundation (PP00P3_170683).

## Competing interests

The authors report no competing interests.

## Supplementary Material

fcab279_Supplementary_Data

## Abbreviations

DTI =: diffusion tensor imaging
DWI =: diffusion-weighted imaging
FWHM =: full-width at half-maximum
MNI =: Montreal Neurological Institute
mRS =: modified Rankin Scale
NIHSS =: NIH Stroke Scale
OR =: odds ratio
RR =: rate ratio
TACS =: total anterior circulation stroke
TIA =: transient ischaemic attack
