# Supplementary material for: Delayed cerebral atrophy after cerebellar stroke: topographical relation and clinical impact
Source: Brain Commun. 2021 Nov 24;3(4):fcab279. doi: 10.1093/braincomms/fcab279 (PMC8643502; doi:10.1093/braincomms/fcab279)
Supplement: fcab279_Supplementary_Data [file fcab279_supplementary_data.zip › Supplementary figures.pdf]

Supplementary figures

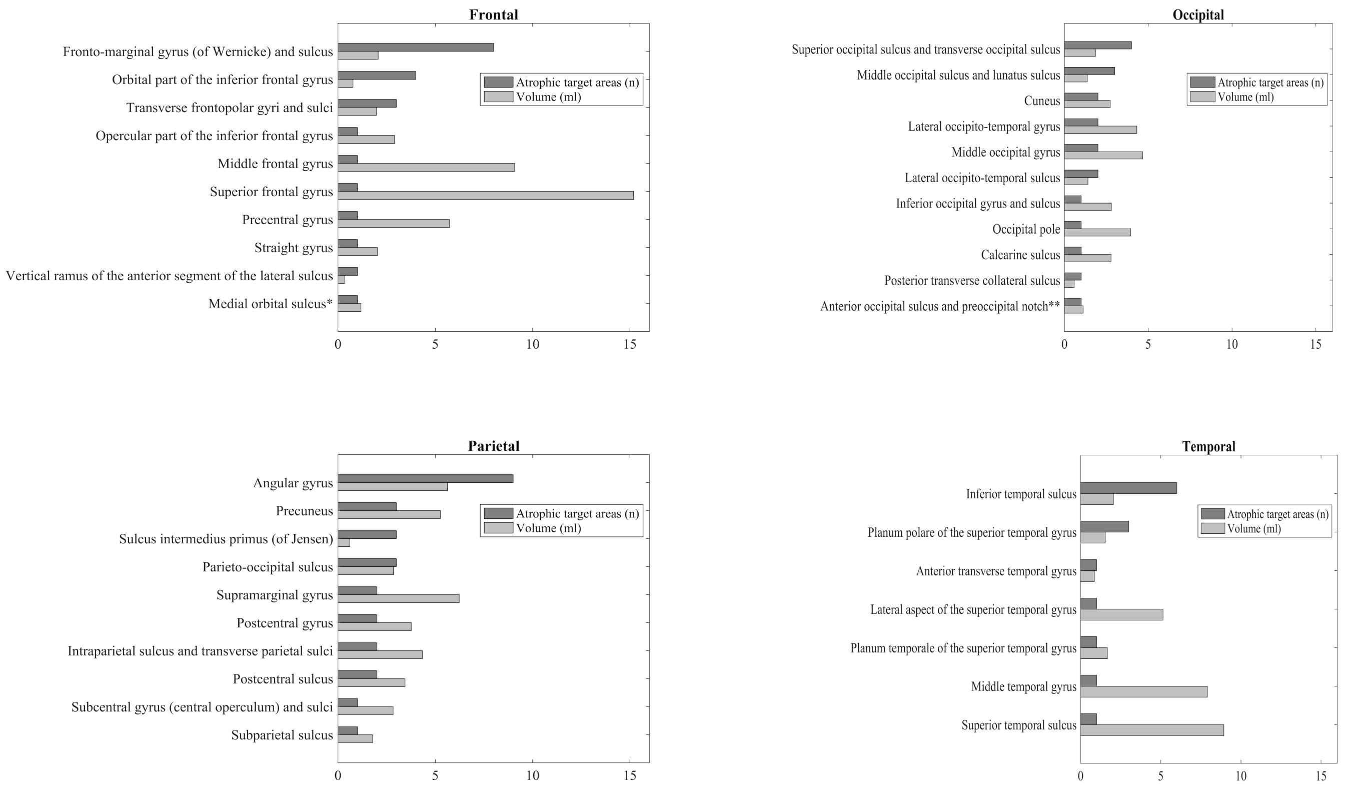

Supplementary Figure 1. **Number of atrophic target areas and baseline cortical volume per target area**

Overview of the number of atrophic target areas and baseline cortical volume per target area. The number of atrophic target areas (n) and the mean baseline cortical volume per target area (ml) are depicted. Atrophic target areas where defined by showing significant change relative to a mixed model fit of reference measures. Stroke volume was manually segmented on MRI-images. Atrophic target areas are classified lobewise in frontal, occipital, parietal and temporal lobes. * Olfactory sulcus, ** Temporo-occipital incisure

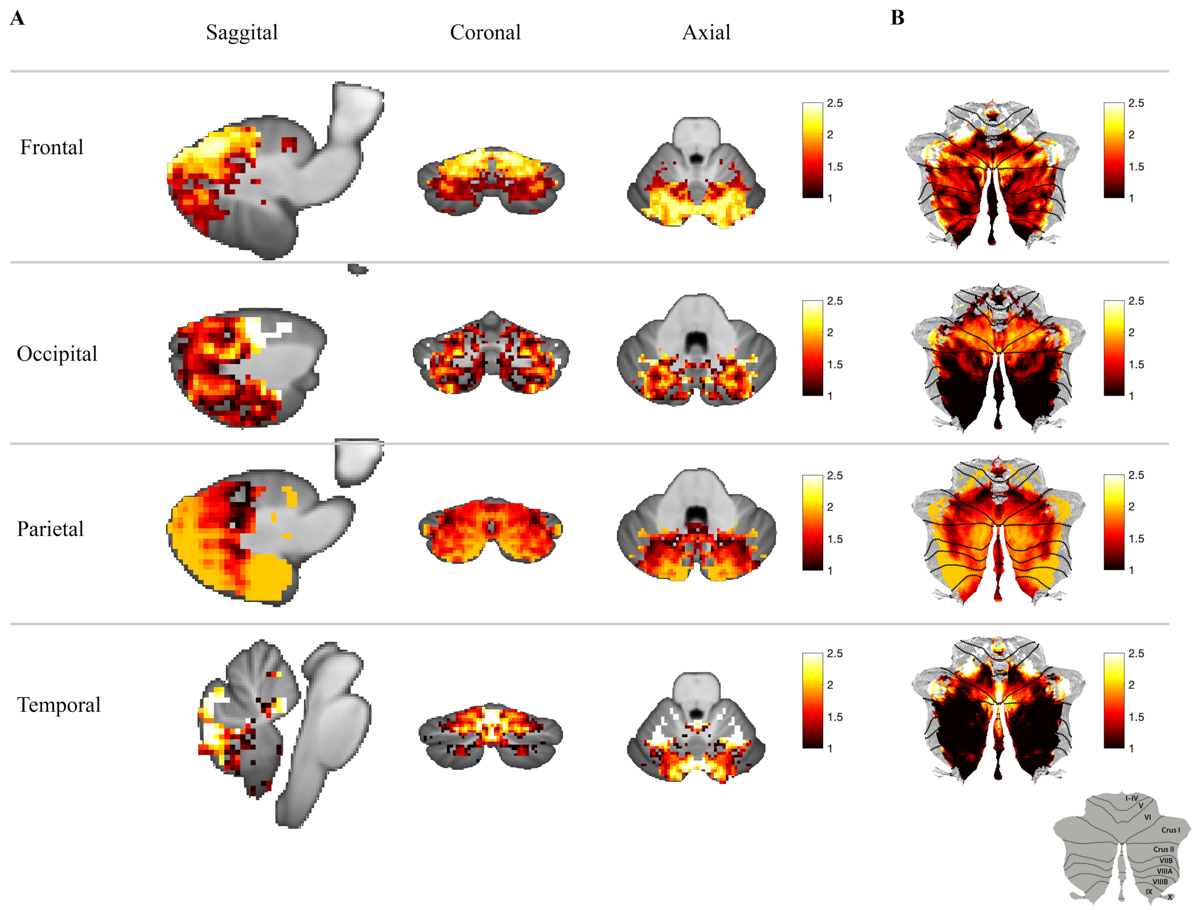

Supplementary Figure 2. **Distribution of stroke as classified by lobewise delayed cortical atrophy**

Distribution of stroke location, depicting the relative risk of a cerebellar voxel being affected in separate lobe-wise groups as compared to the study population. Maps of frontal occipital, parietal and temporal distributions are subsequently shown. The color bar depicts the risk ratio of a voxel being affected relative to the study population mean. **A:** Projected on volumetric anatomical cerebellum-only atlas template.[49] **B:** Projected on cerebellar grey matter flatmap, see lower right for anatomical reference depicting the names of cerebellar lobes.[50]
