# Supplementary material for: Delayed cerebral atrophy after cerebellar stroke: topographical relation and clinical impact
Source: Brain Commun. 2021 Nov 24;3(4):fcab279. doi: 10.1093/braincomms/fcab279 (PMC8643502; doi:10.1093/braincomms/fcab279)
Supplement: fcab279_Supplementary_Data [file fcab279_supplementary_data.zip › Supplementary text & tables.docx]

# Supplementary text and tables

**Supplementary text 1: Violations of the proportionality of effects assumption.**

Disproportionality of several effects was observed for prediction of mRS: univariable: baseline score, gender, stroke location (mRS = 0-5 compared to mRS = 6); multivariable: stroke location, interaction between baseline score and atrophic target areas (mRS = 0-5 compared to mRS = 6)). Furthermore, disproportionality of effects was observed for prediction of NIHSS score in several instances: uni- and multivariable: gender (NIHSS ≥ 5 compared to NIHSS = 0-4); multivariable: stroke location (NIHSS ≥ 5 compared to NIHSS = 0-4 and NIHSS ≥ 4 compared to NIHSS = 0-3).

**Supplementary table 1: Univariable analysis of change in the number of atrophic target areas at last follow-up**

|  | RR | 95% CI | P-value |
| --- | --- | --- | --- |
| Stroke volume | 1.02 | 0.99 - 1.06 | 0.24 |
| Stroke location (bilateral) | 1.43 | 0.66 - 3.08 | 0.35 |
| Side of stroke (left) | 0.79 | 0.35 - 1.79 | 0.56 |
| Baseline cortical volume | 1.15 | 0.96 - 1.37 | 0.11 |
| Age | 0.98 | 0.96 - 1.01 | 0.17 |
| Follow-up time | 0.94 | 0.87 - 1.01 | 0.10 |
| Gender (male) | 0.90 | 0.39 – 2.10 | 0.80 |

Abbreviations: RR= rate ratio; CI= confidence interval

Units: Volume, ml.; Baseline cortical volume, 10 ml.; Age, years; Follow-up time, months.

**Supplementary table 2: Validation of methods: effect of stroke volume on supratentorial atrophy**

| Prediction interval of mixed model estimation | RR | 95% CI | P-value |
| --- | --- | --- | --- |
| 95 % | 1.08 | 1.02 - 1.14 | 0.01 |
| 90 % | 1.06 | 1.01 - 1.11 | 0.03 |
| 80 % | 1.06 | 1.01 - 1.11 | 0.03 |
| 70 % | 1.05 | 1.00 - 1.10 | 0.06 |
| 60 % | 1.05 | 1.00 - 1.10 | 0.049 |
| 50 % | 1.05 | 1.00 - 1.10 | 0.06 |

Abbreviations: RR= rate ratio; CI= confidence interval.

Consistency of the adjusted effect of cerebellar stroke volume on supratentorial cortical atrophy, in patients with bilateral stroke. The analysis was repeated at different prediction intervals of the mixed model estimation of supratentorial cortical volume change.

**Supplementary table 3: Univariable** **analysis of 3-month mRS score**

|  | OR | 95% CI | P-value |
| --- | --- | --- | --- |
| Number of atrophic target areas | 1.07 | 0.85 - 1.28 | 0.52 |
| Stroke location (bilateral) | 2.25 | 0.48 - 11.24 | 0.31 |
| Age | 1.02 | 0.98 - 1.06 | 0.31 |
| Gender (male) | 0.67 | 0.15 - 3.01 | 0.60 |
| Baseline mRS (≥3) | 6.32 | 1.4 - 35.97 | 0.02 |

Abbreviations: OR: odds ratio; CI: confidence interval; mRS: modified Rankin Scale

Units: Age, years.

Unadjusted odds ratios from univariable ordinal regression analysis of 3-month mRS score. All patients had available baseline and follow-up mRS scores.

**Supplementary table 4: Univariable analysis of 3-month NIHSS score**

|  | OR | 95% CI | P-value |
| --- | --- | --- | --- |
| Number of atrophic target areas | 0.91 | 0.59 - 1.27 | 0.62 |
| Stroke location (bilateral) | 2.32 | 0.37 - 13.61 | 0.35 |
| Age | 1.11 | 1.04 - 1.23 | 0.01 |
| Gender (male) | 1.26 | 0.21 - 10.25 | 0.81 |
| Baseline NIHSS (≥6) | 7.62 | 0.65 - 108.32 | 0.10 |

Abbreviations: OR: odds ratio; CI: confidence interval; NIHSS: NIH Stroke Scale

Units: Age, years.

Unadjusted odds ratios from univariable ordinal regression analysis of 3-month NIHSS score. Of 29 Patients, 27 had available baseline and follow-up NIHSS-measurements.

**Supplementary table 5: Multivariable analysis of 3-month NIHSS score**

|  | OR | 95% CI | P-value |
| --- | --- | --- | --- |
| Number of atrophic target areas | 0.77 | 0.42 - 1.23 | 0.33 |
| Stroke location (bilateral) | 2.19 | 0.26 - 18.77 | 0.46 |
| Age | 1.14 | 1.05 - 1.30 | 0.01 |
| Gender (male) | 0.26 | 0.02 - 3.27 | 0.28 |

Abbreviations: OR: odds ratio; CI: confidence interval; NIHSS: NIH Stroke Scale

Units: Age, years.

Adjusted odds ratios from multivariable ordinal regression analysis of 3-month NIHSS score. The patient age is normalized to its mean, corrected effects of stroke location must therefore be interpreted at mean age. Baseline NIHSS was excluded as predictor, as the low number of patients with a high baseline score destabilized the model. Of 29 Patients, 27 had available baseline and follow-up NIHSS-measurements.

**Supplementary table 6: Validation of methods: effect of delayed cerebral atrophy on clinical outcome**

| Prediction interval of mixed model estimation | OR | 95% CI | P-value |
| --- | --- | --- | --- |
| 95 % | 1.34 | 1.05 – 1.76 | 0.02 |
| 90 % | 1.31 | 1.04 – 1.69 | 0.02 |
| 80 % | 1.29 | 1.04 – 1.62 | 0.02 |
| 70 % | 1.28 | 1.04 – 1.59 | 0.02 |
| 60 % | 1.24 | 1.03 - 1.50 | 0.02 |
| 50 % | 1.21 | 1.03 - 1.45 | 0.02 |

Abbreviations: OR= odds ratio; CI= confidence interval.

Consistency of the adjusted effect of the number of atrophic target areas on 3-month mRS in patients with baseline mRS score ³3. The analysis was repeated at different prediction intervals of the mixed model estimation of supratentorial cortical volume change.
